# Pioneering Data Processing for Convolutional Neural Networks to Enhance the Diagnostic Accuracy of Traditional Chinese Medicine Pulse Diagnosis for Diabetes

**DOI:** 10.3390/bioengineering11060561

**Published:** 2024-06-02

**Authors:** Wei-Chang Yeh, Chen-Yi Kuo, Jia-Ming Chen, Tien-Hsiung Ku, Da-Jeng Yao, Ya-Chi Ho, Ruei-Yu Lin

**Affiliations:** 1 Department of Industrial Engineering and Engineering Management, National Tsing Hua University, Hsinchu 30013, Taiwan; wcyeh@ie.nthu.edu.tw (W.-C.Y.); caroline0921@gapp.nthu.edu.tw (R.-Y.L.); 2 Changhua Christian Hospital, Changhua 50051, Taiwan; 3 Department of Power Mechanical Engineering, National Tsing Hua University, Hsinchu 30013, Taiwan; djyao@mx.nthu.edu.tw (D.-J.Y.);

**Keywords:** traditional Chinese medicine (TCM), pulse diagnosis, diabetes, deep learning, pulse waveform analysis, healthcare, ResNet, LeNet

## Abstract

Traditional Chinese medicine (TCM) has relied on pulse diagnosis as a cornerstone of healthcare assessment for thousands of years. Despite its long history and widespread use, TCM pulse diagnosis has faced challenges in terms of diagnostic accuracy and consistency due to its dependence on subjective interpretation and theoretical analysis. This study introduces an approach to enhance the accuracy of TCM pulse diagnosis for diabetes by leveraging the power of deep learning algorithms, specifically LeNet and ResNet models, for pulse waveform analysis. LeNet and ResNet models were applied to analyze TCM pulse waveforms using a diverse dataset comprising both healthy individuals and patients with diabetes. The integration of these advanced algorithms with modern TCM pulse measurement instruments shows great promise in reducing practitioner-dependent variability and improving the reliability of diagnoses. This research bridges the gap between ancient wisdom and cutting-edge technology in healthcare. LeNet-F, incorporating special feature extraction of a pulse based on TMC, showed improved training and test accuracies (73% and 67%, respectively, compared with LeNet’s 70% and 65%). Moreover, ResNet models consistently outperformed LeNet, with ResNet18-F achieving the highest accuracy (82%) in training and 74% in testing. The advanced preprocessing techniques and additional features contribute significantly to ResNet18-F’s superior performance, indicating the importance of feature engineering strategies. Furthermore, the study identifies potential avenues for future research, including optimizing preprocessing techniques to handle pulse waveform variations and noise levels, integrating additional time–frequency domain features, developing domain-specific feature selection algorithms, and expanding the scope to other diseases. These advancements aim to refine traditional Chinese medicine pulse diagnosis, enhancing its accuracy and reliability while integrating it into modern technology for more effective healthcare approaches.

## 1. Introduction

Traditional Chinese medicine (TCM) has long relied on pulse diagnosis as a key method for assessing patient conditions. This ancient technique involves analyzing pulse characteristics to determine the health status of various organs and systems in the body. However, the subjective nature of pulse diagnosis can lead to variations in perception among practitioners, particularly when diagnosing complex conditions like diabetes. To address this challenge, integrating modern technology, such as deep learning algorithms, can help mitigate human errors and enhance diagnostic consistency.

In our endeavor to enhance the accuracy of TCM pulse diagnosis, we have chosen to focus on diagnosing diabetes. Diabetes, a chronic metabolic condition, is characterized by elevated blood glucose levels, leading to potential damage to various organs over time. Globally, there is a concerted effort to curb the escalating rates of diabetes and obesity by 2025. With approximately 422 million people worldwide affected by diabetes, predominantly in low- and middle-income countries, and around 1.5 million deaths each year directly linked to the condition, both the prevalence and incidence of diabetes have steadily risen over recent decades. Hence, our goal is to utilize non-invasive TCM pulse diagnosis to promptly and conveniently diagnose diabetes, facilitating early access to a suitable treatment for patients.

Fu and Lai (1989) presented a system designed to enhance the objectivity of pulse diagnosis within Chinese medicine [1]. The system, consisting of a pressure transducer, an amplifier, and a microcomputer, facilitates the acquisition and processing of data on pulse waveforms. By providing quantifiable and objective pulse characteristics, such as amplitude, rate, and waveform shapes, the system aims to aid practitioners in making more accurate diagnoses based on solid, quantifiable metrics.

Yu et al. (1996) investigated the application of artificial neural networks (ANNs) to categorize pulse signals for analyzing the autonomic nervous system [2]. They used pulse data from a Finapres device, which continuously measures finger arterial pressure waveforms, and trained an ANN with extracted features like pulse rate and amplitude. The study demonstrated the feasibility and potential of ANNs in classifying pulse signals, indicating a promising approach to assess autonomic function through pulse analysis.

Wang and Lin (1998) developed a detection system to acquire pulse signals from the radial artery and applied power–spectral analysis to analyze these signals [3]. They focused on extracting frequency-domain features and comparing the power–spectral density between normal and abnormal pulse signals. The findings revealed significant differences in the spectral characteristics of various pulse types, suggesting that power–spectral analysis could serve as a valuable quantitative tool for pulse diagnosis in TCM.

Yoon et al. (2000) explored a method for classifying pulse types by varying the contact pressure during the pulse-taking process [4]. They utilized a device to record pulse waves under light, medium, and heavy pressure and analyzed these waves to extract features such as amplitude, width, and area. Using discriminant analysis based on these features, the study achieved an 80% accuracy rate in classifying pulse types, underscoring the effectiveness of adjusting the contact pressure to enhance the diagnostic accuracy in pulse analysis.

In 2006, Wang proposed a quantitative system based on Bayesian networks (BNs) [5] to map 407 pulse waves from 298 patients and 109 healthy volunteers to pulse types (moderate or rapid and floating or sinking) in TCM (Wang & Cheng, 2006). The proposed system demonstrates the potential of integrating modern technology into traditional Chinese pulse diagnosis to improve its objectivity and accuracy.

Bote-Curiel et al. (2019) [6] presented a methodology focused on utilizing big data and deep learning technologies for disease diagnosis. The study highlighted the transformation of medical data into structured formats and the use of convolutional neural network (CNN) models to improve diagnostic accuracy. While demonstrating significant advancements over traditional methods, limitations were noted, particularly concerning data collection capabilities and model interpretability.

Mirpouya Mirmozaffari et al. (2021) [7] explored optimization techniques combined with machine learning for various industries, employing DEA models to assess the efficiency in cement companies. The results indicated that the highest efficiency scores were obtained with the FDH model and the highest clustering accuracy was obtained with the K-means algorithm. However, challenges in data collection and model interpretation were acknowledged.

Roy et al. (2022) [8] investigated the application of supervised machine learning (SML) and artificial intelligence (AI) in digital healthcare. The study emphasized the role of SML in disease detection and classification, utilizing advanced techniques like deep neural networks and natural language processing. While highlighting the potential of SML in healthcare, challenges such as real-world clinical trial limitations and the need for extensive training and updates were discussed.

Shiva Maleki Varnosfaderani et al. (2024) [9] highlight the advancements in AI, particularly deep learning, for medical image analysis, with a specific focus on bone fracture identification and evaluation within musculoskeletal radiology. Their work emphasizes the improved diagnostic accuracy and efficiency offered by these AI methods, often surpassing human clinicians and already available in commercial products for clinical integration. Despite these achievements, challenges like missed fractures, escalating radiologist workloads, and variations in image interpretation persist, underscoring the ongoing need for AI solutions. The review by Maleki Varnosfaderani et al. aimed to delve into the current performance of AI in fracture identification and evaluation, shed light on existing commercial offerings, and address the limitations and future prospects in this evolving field.

Tieu et al. (2024) [10] describe the advancements in AI, specifically deep learning, for the analysis of medical images, focusing on bone fracture identification and evaluation in musculoskeletal radiology. These AI methods offer improved diagnostic accuracy and efficiency, comparable to or better than human clinicians, with commercial products already available for integration into clinical workflows. However, challenges such as missed fractures, increasing radiologist workloads, and variations in image interpretation persist, prompting the need for AI solutions. The review aimed to explore the current performance of AI in fracture identification and evaluation, highlight commercial products, and discuss limitations and future directions in the field.

Recent advancements in medical imaging and artificial intelligence have opened new avenues for integrating technology into traditional medical practices. In particular, Residual Neural Networks (ResNets) have demonstrated exceptional explanatory power when applied to medical image processing, showing promise in diagnosing conditions such as lung tumors, skin diseases, breast diseases, and brain disorders (Xu et al., 2023) [10]. Furthermore, Deng et al. (2022) [11] proposed a convolutional neural network-based classification method using an improved Inception–ResNet network for diagnosing viral pneumonia in elderly patients through lung CT images.

Based on these advancements, our study aimed to integrate ten years’ worth of pulse diagnosis instrument data from our traditional Chinese medicine (TCM) department. We propose a novel approach to preprocessing TCM pulse data to be input into a CNN for comprehensive data analysis, aiming to identify pulse characteristics associated with various diseases. By utilizing our proposed data processing method and leveraging the power of ResNet models, we aimed to enhance the diagnostic capabilities of modern TCM pulse diagnosis instruments and improve the accuracy of diabetes diagnosis.

The significance of this research lies in integrating traditional medical practices into cutting-edge technology to provide more comprehensive and effective patient care. With the increasing prevalence of diabetes in Asia, there is an urgent need for enhanced diagnostic methods and early intervention strategies.

In this paper, we propose a novel data processing method to convert TCM pulse data into a format suitable for a CNN (such as a ResNet), aiming to improve the accuracy of diabetes diagnosis through pulse analysis. We outline our methodological framework, data sources, data processing methods, and results from various model experiments. By demonstrating the potential of integrating deep learning algorithms into traditional Chinese medicine pulse diagnosis, we aim to contribute to improved healthcare outcomes and pave the way for further exploration in the integration of traditional Chinese medicine and modern healthcare.

Our proposed approach involves several key steps. Firstly, we collected a decade’s worth of pulse diagnosis instrument data from the Department of TCM at Changhua Christian Hospital, laying the foundation for our analysis. Next, we developed a new feature extraction and data preprocessing pipeline to transform the raw pulse data into a format compatible with CNN architectures, which was crucial for ensuring the effectiveness of the subsequent analysis.

Once we had completed the data preprocessing, we utilized CNN models, such as ResNets, employed methods for feature extraction, and adjusted model parameters to identify the optimal model configuration for analyzing pulse characteristics and identifying patterns associated with diabetes. The deep architecture and ability to capture complex features of CNNs make them well-suited for this task. By fine-tuning ResNet models on our processed pulse data, we aimed to enhance the accuracy and reliability of diabetes diagnosis.

Furthermore, our study stands out in utilizing TCM pulse data for analysis, which is not common in the literature. This integration of ancient Chinese medicine into modern science, coupled with the trust in and reliance on traditional Chinese medicine among Asian populations, may increase their willingness to participate in medical examinations. Such proactive healthcare initiatives have the potential to mitigate the rising prevalence of diabetes.

This article is organized as follows. The methodology is introduced in Section 2. Section 3 addresses the use of a CNN in TCM pulse diagnosis with the proposed data processing techniques. Our conclusions are presented in Section 4. Finally, Section 5 describes future work.

## 2. Methodology

This section delves into the methodology adopted for our study, which revolves around the utilization of convolutional neural networks (CNNs), a prominent subset of artificial neural networks (ANNs). CNNs, founded on ANN principles, serve as the cornerstone of our investigation. Specifically, our focus is directed toward the comprehensive evaluation of two distinguished CNN architectures: LeNet and ResNet. To contextualize our approach, we commence with an elucidation of the fundamental workings of ANNs. Following this, we embark on a detailed examination of the LeNet and ResNet models, dissecting their individual attributes and significant contributions within the realm of deep learning.

Within this methodological framework, we pay careful attention to data management, encapsulating all of the data handling processes, including collection, preprocessing, and feature extraction. Recognizing the adage of “garbage in, garbage out”, we underscore the criticality of stringent data management practices, which are especially pertinent when preparing data for CNN models. The integration of traditional Chinese medicine (TCM) pulse diagnosis into advanced data analytics necessitates an unwavering commitment to data integrity and confidentiality. From the methodical acquisition of data in clinical environments to the rigorous preprocessing procedures that are essential to furnishing high-quality data inputs for machine learning algorithms, each stage is meticulously designed to optimize the precision and efficacy of the diagnostic models. The subsequent sections delve deeper into these pivotal stages, commencing with an exploration of the data collection process.

### 2.1. ANNs

An ANN, also referred to as a multi-layer perceptron, is a network model that comprises multiple nodes and connections [12]. These nodes, analogous to neurons in the human brain, facilitate the transmission of information by forming connections between different layers. In an ANN, each neuron in a layer is connected to every neuron in the subsequent layer, although connections do not exist between neurons within the same layer.

As networks become deeper and gain more hidden layers, they evolve into deep neural networks (DNNs), which are capable of addressing increasingly complex problems. The depth of these networks, as noted by researchers such as LeCun et al. (2015) [13] and Goodfellow et al. (2016) [14], allows for enhanced problem-solving capabilities, reflecting significant advancements in the field of neural networks.

### 2.2. LeNet

LeNet, also known as LeNet-5, is one of the earliest and simplest CNN architectures, having a profound impact on the field of deep learning. Developed by Yann LeCun and his team in 1998, LeNet was primarily designed for the task of handwritten digit recognition, such as identifying characters on checks [15].

**Figure 1:**
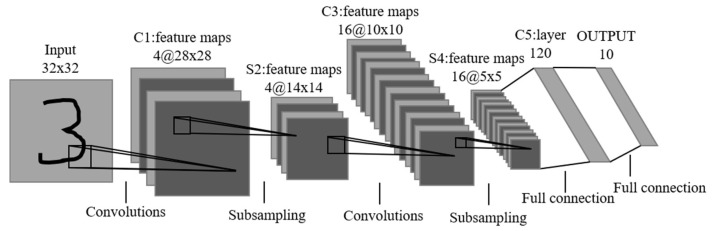
LeNet-4 [14,15].

As shown in Figure 1, the architecture of LeNet is distinguished by its sequence of convolutional layers, followed by subsampling (pooling) layers, and concluding with fully connected layers. The essential operation within a convolutional layer of LeNet can be mathematically described by the equation:

(1) y=F(W ∗ x+b)

where x denotes the input, *y* represents the output, F is the mapping function, and W and b are the weights and biases, respectively.

The integration of LeNet into this study is significant as it serves as a fundamental model in the history of neural networks. The design principles of LeNet, especially the use of convolutional layers and subsampling, have been pivotal in inspiring further developments in neural network architectures. While LeNet itself is not the primary focus here, its inclusion provides crucial context for understanding the progression of neural network architectures and their diverse applications, particularly in complex tasks like diabetes prediction. Moreover, as a benchmark model, LeNet provides a basis for comparison with more recent architectures such as ResNet, which is also examined in this study. Analyzing LeNet’s mathematical foundations enhances our understanding of its influential role in shaping the development of deep learning, as it represents one of the simplest forms of CNN.

### 2.3. ResNet

In recent years, the field of deep learning has experienced significant advancements, particularly in domains such as image recognition and natural language processing. Among the various neural network architectures that have been developed, ResNets have garnered a substantial amount of attention due to their exceptional performance. This section investigates the selection of ResNet for diabetes prediction, elucidating its architectural strengths and specific applications within this context.

ResNet’s role in diabetes prediction is pivotal due to its capacity to overcome common deep learning challenges, such as the vanishing and exploding gradient problems. The introduction of residual blocks into ResNet allows the network to learn identity mappings smoothly, effectively addressing the vanishing gradient issue. These blocks enhance the network’s ability to perform deep feature extraction, which is crucial for accurately predicting diabetes.

The function of a residual block in ResNet can be described mathematically as

(2) $$y=F\left(x,\left\{W_{i}\right\}\right)+x$$

where x is the input, y is the output, $F\left(x,\left\{W_{i}\right\}\right)$ represents the mapping function of the residual branch, and $\left\{W_{i}\right\}$ are the weights associated with the residual branch. This formulation facilitates efficient learning and gradient optimization, thereby enhancing the overall model performance.

Developed by Kaiming He and his team in 2015 [16], ResNet revolutionized deep learning by improving the gradient flow through residual blocks. For diabetes prediction, this architectural feature allows the model to capture complex patterns and subtle features associated with diabetes risk factors. The deep structure of ResNet is particularly beneficial in discerning intricate details within diabetes-related data.

The application of ResNet in this research involved fine-tuning pre-trained models to adapt to the specific nuances of diabetes data. Adjustments were made to the model’s depth and configuration to better suit the unique characteristics of the dataset and optimize the model’s predictive accuracy for diabetes.

This section delves into the nuances of the application of ResNet in predicting the likelihood of diabetes. By fine-tuning the model and experimenting with its configuration, we aimed to demonstrate how ResNet’s unique capabilities can be effectively utilized in healthcare, offering new perspectives on predictive modeling for diabetes and potentially enhancing diagnostic approaches.

### 2.4. Dataset and Changhua Christian Hospital

The dataset used in this study resulted from multidisciplinary collaboration with doctors from the Department of Traditional Chinese Medicine (TCM) at Changhua Christian Hospital (CCH). Established in 2006, the department is dedicated to promoting the holistic spirit of TCM, developing a comprehensive TCM inheritance system, delivering high-quality TCM healthcare services, and advancing TCM research and innovation.

The data were collected from the outpatient pulse diagnosis database of Changhua Christian Hospital, covering the period from 2010 to 2020. The dataset includes data on patients who underwent a pulse diagnosis and heart rate variability (HRV) examination in the TCM outpatient department. The data acquisition was performed using the ANS Watch device, an HRV monitor approved by the Taiwan Food and Drug Administration (License No. 001525).

The clinical research dataset comprises data on 1001 participants, consisting of 616 individuals diagnosed with diabetes by Western medical practitioners and 385 individuals confirmed by Western physicians to be free of diabetes. To ensure the diversity of the data and capture various pulse characteristics, three different types of pulse wave data were collected for each subject (shallow, medium, and deep).

To facilitate model training and evaluation, the dataset was divided into training and testing sets using a 70% to 30% ratio. This split ensured that the model’s performance would be assessed on unseen data, providing a more realistic estimate of its generalizability when encountering new cases. A 70% to 30% ratio is commonly used in machine learning in order to strike a balance between having sufficient data for training the model and evaluating its performance on an independent test set.

By leveraging this diverse and comprehensive dataset, we aimed to develop deep learning models that can enhance the accuracy of traditional Chinese medicine pulse diagnosis for diabetes. The collaboration with TCM doctors from Changhua Christian Hospital brought valuable expertise and insights to the research, ensuring its clinical relevance and potential for real-world application.

### 2.5. Data Collection 

This study involved the acquisition of data from the outpatient pulse diagnosis database at Changhua Christian Hospital, covering the period from 2010 to 2020. The dataset comprised the records of patients who had undergone pulse diagnosis and heart rate variability (HRV) assessments in the traditional Chinese medicine (TCM) outpatient department utilizing the ANS Watch device, which is certified by the Taiwan Food and Drug Administration (License No. 001525).

Owing to the retrospective design of the study, the requirement for informed consent was obviated, enabling the utilization of existing medical records without direct patient interaction. To safeguard patient confidentiality and ensure the ethical handling of the data, all identifiable information was removed from the records. Each record was subsequently assigned a unique research code, a measure that was pivotal in maintaining the data’s integrity while facilitating comprehensive analysis.

The records were systematically categorized according to ICD-10/ICD-9 diagnostic codes. This categorization facilitated a structured analysis by aligning the records with prevalent diseases in both TCM and Western medical practices. Such an alignment enabled the targeted examination of treatment outcomes before and after TCM interventions across various disease groups, thereby enhancing the study’s clinical relevance.

Furthermore, the study sought to refine the diagnostic precision of TCM by integrating data obtained from traditional pulse diagnosis instruments. This integration aimed to merge conventional diagnostic methodologies with contemporary analytical techniques, thereby enriching the robustness and applicability of the research findings. All data collection procedures were conducted in a non-invasive manner, strictly adhering to minimal risk protocols to ensure patient safety and uphold rigorous ethical standards in medical research. This methodological rigor highlights the study’s commitment to advancing the diagnostic capabilities of TCM within the framework of modern medical ethics and data protection regulations.

### 2.6. Ethical Considerations

Ethical considerations were paramount in this study in order to ensure privacy and confidentiality for all participants. A thorough de-identification process was employed, with each participant assigned a unique code in order to maintain anonymity. The security of the data was upheld by storing them on password-protected computers and in physically secured cabinets accessible only to designated research staff.

All research personnel, including the principal investigator and co-investigators, committed to strict confidentiality protocols in order to safeguard participant information. Electronic data were protected with antivirus software to prevent unauthorized access and ensure the integrity of the research data throughout the study. These measures reflect the high standards of data protection and ethical conduct adhered to in this research, emphasizing the safeguarding of participant privacy and the security of research data.

### 2.7. Data Preprocessing 

**Figure 2:**
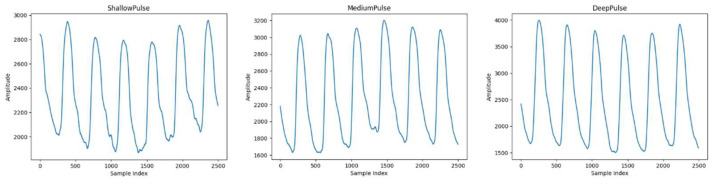
Pulse wave (with a diagnosis of diabetes).

**Figure 3:**
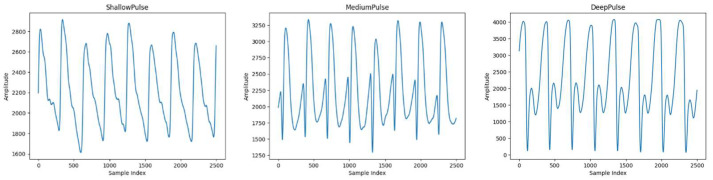
Pulse wave (without a diagnosis of diabetes).

The pulse waveforms presented in Figure 2 and Figure 3 were collected using the ANS Watch device. With each measurement taken using the ANS Watch device on a single subject, three types of pulses—shallow pulses, medium pulses, and deep pulses—can be obtained. The following descriptions correspond to the different pulse types.

Floating pulses can be easily detected with a light touch and weaken slightly under heavy pressure without becoming empty. The main indications are surface manifestations, which are also indicative of deficiency patterns.

Regarding deep pulses, a light touch yields no response. Deep pulses require heavy pressure to be felt. The main condition is an internal syndrome. A strong and sunken pulse indicates an internal excess, while a weak and sunken pulse indicates an internal deficiency.

Figure 2 depicts the pulse of a patient diagnosed with diabetes according to Western medicine, while Figure 3 illustrates the pulse waveform of a patient confirmed to be free of diabetes according to a Western medical examination.

**Figure 4:**
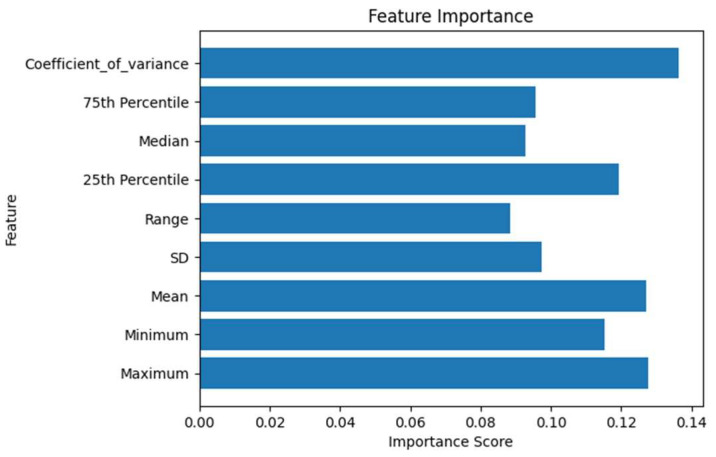
Feature Importance Chart.

At this stage, we first identified some basic statistical measurements of the three pulse waveforms for each participant, including the coefficient of variance, the 75th percentile, the median, the 25th percentile, the range, the standard deviation (SD), the mean, the minimum, and the maximum. Subsequently, we employed the widely used machine learning model XGBoost and used the aforementioned statistical measurements as features for model learning. By employing a binary classification approach (0, no diabetes; 1, diabetes), we conducted a preliminary classification. In this attempt, the model achieved an accuracy of 65%. Building upon this, we performed feature importance analysis for the XGBoost model, resulting in the chart shown in Figure 4. 

Based on the histogram of feature importance (Figure 4), we selected the features with the top three scores—coefficient of variance, maximum, and mean—to be emphasized in weight during the training of the ResNet model.

### 2.8. Feature Extraction

In order to effectively analyze the pulse waveforms and identify distinguishing characteristics between diabetic and non-diabetic individuals, we employed a comprehensive data preprocessing and feature extraction approach. This process involved the calculation of several statistically significant metrics that capture key aspects of the pulse waveforms.

Firstly, we computed the coefficient of variance (CV) for each pulse waveform. The CV is a measure of dispersion that quantifies the variability in data points around the mean. By calculating the CV, we aimed to gain insights into the relative variability in pulse waveforms between the diabetic and non-diabetic groups. A higher CV value indicates greater variability, while a lower CV value suggests more consistency in the pulse waveform.

Secondly, we extracted the maximum value from each pulse waveform. The maximum value represents the highest amplitude or peak reached by the waveform. Analyzing the maximum value allows us to investigate potential differences in the peak characteristics of pulse waveforms between diabetic and non-diabetic individuals. Significant variations in the maximum value could indicate distinct patterns or abnormalities associated with diabetes.

Thirdly, we calculated the mean value of each pulse waveform. The mean value provides a measure of the average amplitude or intensity of the waveform. By comparing the mean values between the two groups, we sought to identify any systematic differences in the overall pulse intensity. Deviations in the mean value could suggest underlying physiological changes related to diabetes.

In addition to these metrics, we placed special emphasis on the first peak of the pulse waveform. Traditional Chinese medicine (TCM) acknowledges that the first peak may exhibit significant differences between diabetic and non-diabetic individuals. Drawing from this understanding, we specifically focused on analyzing the characteristics of the first peak, such as its amplitude, width, and shape. By closely examining the first peak, we aimed to uncover potential diagnostic markers that could aid in distinguishing between the two groups.

Through this comprehensive data preprocessing and feature extraction approach, we aimed to capture meaningful information from the pulse waveforms that could contribute to the development of more accurate diagnostic methods in TCM. By considering a combination of statistical metrics and domain-specific knowledge, we sought to identify the most discriminative features that could enhance the differentiation between diabetic and non-diabetic individuals based on their pulse characteristics.

The extracted features, including the coefficient of variance, maximum value, mean value, and first peak characteristics, formed the basis for the subsequent analysis and modeling. These features were carefully selected to provide a multifaceted representation of the pulse waveforms, enabling us to explore their potential in improving the accuracy of diabetes diagnosis in the context of TCM.

## 3. CNNs in TCM Pulse Diagnosis Using the Proposed Data Processing Techniques

In this section, we present the dataset, experimental setup, and results obtained from our study on enhancing the accuracy of TCM pulse diagnosis for diabetes using CNNs.

### 3.1. Experimental Environment and Configuration

To ensure the reproducibility of our experiments and provide transparency regarding the computational resources employed, we conducted all experiments using a consistent hardware and software configuration. The details of the computing environment are as follows:

1. Processor: 11th Gen Intel(R) Core(TM) i7-1165G7 @ 2.80GHz. This processor is manufactured by Intel Corporation, headquartered at 2200 Mission College Blvd., Santa Clara, CA 95054, USA. This processor provides a balance between performance and power efficiency. Its base clock speed of 2.80 GHz allows for the efficient execution of computationally intensive tasks, such as training deep learning models.
2. Memory: 16.0 GB RAM. The system was equipped with 16.0 GB of Random Access Memory (RAM), which is sufficient for handling the memory requirements of the deep learning models and the associated data processing tasks. This amount of memory ensured the smooth execution of the experiments and that we would not encounter memory constraints.
3. Operating System: Windows 10. We utilized the Windows 10 operating system, which offers a stable and user-friendly environment for conducting experiments. Windows 10 supports various software tools and libraries commonly used in machine learning and data science workflows. Its compatibility with a wide range of hardware configurations made it a suitable choice for our experimental setup.
4. Python version: 3.10.12. Python is a widely used programming language in the fields of machine learning and data science. We utilized Python version 3.10.12, which is a stable release offering a range of features and improvements. This version ensured compatibility with the deep learning framework and other dependencies used in the study.
5. Deep learning framework: PyTorch 2.2.1+cu121. PyTorch is a popular open-source deep learning framework known for its dynamic computational graphs and ease of use. We employed PyTorch version 2.2.1 with CUDA 12.1 support, enabling the utilization of GPU acceleration for training and inference. This combination of PyTorch and CUDA ensured the optimal performance of and compatibility with the available hardware.

By providing detailed information about the computing infrastructure used in our experiments, we aim to facilitate the reproducibility of our work and enable other researchers to build upon our findings. These specifications serve as a reference point for setting up a similar environment and ensure that the results can be replicated under comparable conditions.

### 3.2. Model Hyperparameters

This study employed two CNN architectures, LeNet and ResNet, to evaluate the effectiveness of our data preprocessing approach and the integration of new features. Specifically, we assessed two variants of the ResNet18 model (the standard ResNet18 model and ResNet18-F) and two LeNet models (LeNet and LeNet-F). The models with F in the name incorporate the proposed advanced data preprocessing techniques and additional features. The ResNet and LeNet architectures were chosen for their distinct characteristics and advantages in pulse waveform analysis. ResNet, known for its deep architecture with residual connections, excels in capturing intricate patterns and long-range dependencies in pulse data. On the other hand, LeNet, with its simpler architecture, is efficient in extracting basic features and is particularly suitable for tasks with limited computational resources. The key parameters considered in the CNN included the following:

- Batch size (the number of samples used in each training iteration). The batch size has a significant impact on the training speed and memory usage. The batch sizes used were 64 for LeNet and LeNet-F and 128 for ResNet18 and ResNet18-F;
- Learning rate (the step size at which the model’s weights are updated). An initial learning rate of 0.001 was used for all models;
- Number of epochs (the total number of traversals of the training dataset). To avoid overfitting, we monitored the number of epochs closely and implemented early stopping when the validation performance no longer improved. The number of epochs, combined with the batch size, determines the total number of weight updates during training. LeNet and LeNet-F were trained for 1000 epochs, while ResNet18 and ResNet18-F were trained for 1500 epochs;
- Weight decay was introduced as a regularization method to prevent overfitting. We started with a small value (1 × 10^−4^) for weight decay. Adjustments were made based on the model’s performance in order to optimize regularization;
- Dropout (a technique that promotes independence among neurons). Dropout rates of 0.2 and 0.3 were tested in order to find the optimal setting for enhancing the model’s robustness.

The optimization of each model was conducted through the meticulous tuning of hyperparameters and with the aim of maximizing performance within the given architectural constraints. We initiated our exploration with the learning rate, the foundational and critical hyperparameter. We commenced with a conservative value of 0.001 and experimented with learning rate decay strategies, such as exponential decay and step decay, to enhance the model’s convergence. In addition, it’s worth noting that we referenced the simplified swarm optimization (SSO) algorithm proposed by Yeh to tune the CNN hyperparameters [17].

By comparing the performance of ResNet18 and ResNet18-F, as well as the performance of LeNet and LeNet-F, this study aimed to evaluate the effectiveness of the proposed data preprocessing approach and the impact of incorporating new features on the models’ ability to accurately predict diabetes based on pulse waveform data. The inclusion of ResNet18-F and LeNet-F allowed for a direct assessment of the benefits gained from the enhanced data preprocessing techniques and the additional features, providing insights into their contribution to improving the models’ predictive power.

Through a comparative analysis of LeNet, LeNet-F, ResNet18, and ResNet18-F, the study aimed to identify the most suitable deep learning architecture and preprocessing approach for the prediction of diabetes based on pulse waveform data. The findings may contribute to the development of more accurate and reliable diagnostic tools in the field of traditional Chinese medicine.

### 3.3. Detailed Analysis of Hyperparameter Tuning

Hyperparameter tuning is a crucial step in optimizing the performance of machine learning models, including convolutional neural networks (CNNs) like LeNet and ResNet. In this study, we performed hyperparameter tuning in order to maximize the performance of our models within the given architectural constraints.

One of the key hyperparameters we focused on was the learning rate, which determines the step size at which the model’s weights are updated during training. We started with a conservative initial learning rate of 0.001 for all models. However, we recognized the importance of fine-tuning this parameter to achieve optimal convergence and avoid issues like overfitting and underfitting. To do so, we experimented with various learning rate decay strategies, such as exponential decay and step decay. By adjusting the learning rate decay strategy and observing its effect on model convergence, we were able to identify the most suitable approach for each model architecture.

Another critical hyperparameter we tuned was the batch size, which defines the number of samples used in each training iteration. The choice of batch size significantly impacts the training speed and memory usage. We carefully selected batch sizes of 64 for LeNet and LeNet-F and 128 for ResNet18 and ResNet18-F based on considerations of computational resources and model convergence. By experimenting with different batch sizes, we aimed to strike a balance between training efficiency and model performance.

Additionally, we optimized the number of epochs, which refers to the total number of traversals of the training dataset. To prevent overfitting, we closely monitored the number of epochs and implemented early stopping when the validation performance no longer improved. LeNet and LeNet-F were trained for 1000 epochs, while ResNet18 and ResNet18-F were trained for 1500 epochs. This careful monitoring and adjustment of the number of epochs helped ensure that our models were adequately trained without overfitting to the training data.

Furthermore, we considered regularization techniques such as weight decay and dropout to prevent overfitting. Weight decay, initialized with a small value of 1 × 10^−4^, helped control the complexity of the models by penalizing large weight values. Dropout, with dropout rates of 0.2 or 0.3, promoted independence among neurons and improved the model’s robustness. By fine-tuning these regularization parameters, we aimed to strike a balance between model complexity and generalization performance.

In summary, the hyperparameter tuning process involved careful experimentation and the optimization of key parameters such as learning rate, batch size, number of epochs, weight decay, and dropout. By systematically adjusting these hyperparameters and evaluating their impact on model performance, we were able to optimize the performance of our CNN models for the accurate prediction of diabetes based on pulse waveform data.

### 3.4. Comparative Analysis of Model Performance and Impact of Advanced Data Preprocessing

The performance comparison of the different models, as shown in Table 1, reveals the potential advantages of the ResNet architecture over LeNet and the benefits of incorporating advanced data preprocessing techniques and additional features.

Comparing LeNet and LeNet-F, we observe that LeNet-F achieves slightly higher training and test accuracies (73% and 67%, respectively) compared with LeNet (70% and 65%, respectively). This improvement can be attributed to the incorporation of advanced data preprocessing techniques and additional features into LeNet-F. These enhancements enable LeNet-F to better capture and utilize diabetes-related characteristics, leading to improved predictive performance.

The results also demonstrate that the ResNet models consistently outperform their LeNet counterparts. The deep residual structure of ResNet helps to alleviate the vanishing gradient problem, enabling the construction of deeper network architectures. This allows the ResNet models to more effectively learn and represent complex features, enhancing their ability to capture abstract characteristics relevant to diabetes prediction.

Among the ResNet variants, ResNet18-F achieved the highest accuracy, with an impressive 82% training accuracy and 74% test accuracy. This improvement can be attributed to the incorporation of advanced data preprocessing techniques and additional features. By leveraging these enhancements, ResNet18-F can more effectively capture and utilize diabetes-related characteristics, leading to improved predictive performance.

The discrepancy in accuracy between ResNet18-F and the standard ResNet18 model suggests that the feature engineering strategies employed in ResNet18-F play a crucial role in its superior performance. The combination of advanced preprocessing techniques and carefully selected features makes ResNet18-F particularly well-suited for the task of diabetes prediction based on pulse waveform data.

Also, from the above, we can observe that the more complex structure leads to higher accuracy, particularly when combined with the proposed data processing method. This indicates that the advanced preprocessing techniques and additional features have a more pronounced positive impact on models with higher complexity, such as ResNet18-F.

It is important to note that all models exhibit higher training accuracy values than test accuracy values, indicating a potential overfitting issue. This can be attributed to the limited sample size used in the study.

In summary, the results highlight the advantages of the ResNet architecture over LeNet and the benefits of incorporating advanced data preprocessing and feature selection techniques. The comparison between LeNet and LeNet-F demonstrates the positive impact of these enhancements on model performance. These findings contribute to the development of more accurate and reliable diagnostic tools in the field of traditional Chinese medicine, particularly for diabetes prediction.

## 4. Conclusions 

This study has shed light on the potential of advanced data processing techniques to revolutionize traditional Chinese medicine pulse diagnosis by integrating it into cutting-edge deep neural network models like ResNet architectures. Our findings underscore the importance of embracing technological advancements to enhance healthcare practices, particularly in the diagnosis of diseases such as diabetes. A key differentiator of our research lies in our close collaboration with hospitals, enabling access to vast and comprehensive datasets, as well as the expertise of physicians in traditional Chinese medicine. By leveraging these techniques, we can bridge the gap between ancient medical traditions and modern healthcare, paving the way to more accurate and personalized diagnostic approaches.

## 5. Future Work

Looking ahead, our research suggests several actionable insights for managers and organizations seeking to leverage advanced data processing techniques in healthcare. These include the following:

1. Investment in technology: Organizations should invest in research and development to explore and adopt advanced data processing techniques. By staying at the forefront of technological innovation, healthcare providers can improve diagnostic accuracy and patient outcomes;
2. Training and education: Managers should prioritize training programs to equip healthcare professionals with the skills and knowledge needed to effectively utilize advanced data processing techniques. This includes training on signal processing algorithms, deep learning models, and the interpretation of diagnostic results;
3. Collaboration and partnerships: Organizations should foster collaboration with technology firms, research institutions, and healthcare practitioners to co-develop innovative solutions. Collaborative efforts can accelerate the development and implementation of advanced diagnostic tools;
4. Regulatory compliance and data security: Managers must ensure compliance with regulatory standards and prioritize data security and privacy in the development and deployment of healthcare technologies. This includes adherence to data protection regulations such as HIPAA and GDPR;
5. Continuous improvement: Organizations should adopt a culture of continuous improvement, regularly evaluating and refining diagnostic algorithms based on real-world data and feedback from healthcare professionals. This iterative approach ensures that diagnostic tools remain relevant and effective over time.

By embracing these insights and implementing strategies to leverage advanced data processing techniques, managers and organizations can drive innovation in healthcare, improve diagnostic accuracy, and, ultimately, enhance patient care.

## Figures and Tables

**Table 1:** Performance Comparison of Different Models.

Model	Training Accuracy (%)	Testing Accuracy (%)
LeNet	70	65
LeNet-F	73	67
ResNet18	73	69
RseNet18-F	82	74

## Data Availability

Data is unavailable due to privacy or ethical restrictions.
